# Breast reconstruction in supernumerary breast and ectopic breast tissue in Poland’s syndrome

**DOI:** 10.1016/j.jpra.2026.05.008

**Published:** 2026-05-08

**Authors:** Hüseyin Emre Ulukaya, Murat Doğuş Çerikan, Egehan Güngörmez, Mehmet Fatih Çamlı, Selami Serhan Şirvan, Kamuran Zeynep Sevim

**Affiliations:** a University of Health Sciences, Şişli Hamidiye Etfal Research and Training Hospital, Istanbul, Turkiye; b Biruni University, Faculty of Medicine, Istanbul, Turkiye

**Keywords:** Breast anomaly, Breast reconstruction, Nipple-areola complex preservation, Parenchymal flap, Poland’s syndrome

## Abstract

**Background:**

Poland’s syndrome is a rare congenital condition characterized by anterior chest wall malformations and, frequently, ipsilateral upper limb deformities. Treatment strategies vary depending on severity and may involve complex reconstructive approaches.

**Case presentation:**

We present the case of a 20-year-old female with Poland’s syndrome who exhibited left-sided supernumerary nipple and significant breast asymmetry. Clinical examination revealed two vertically aligned nipples and accessory breast tissue 6 cm lateral to the midclavicular line. The patient also presented with agenesis of the left 4th, 5th, and 6th ribs. Surgical management included excision of ectopic breast tissue and transposition of glandular tissue as a centrally based dermoglandular flap. Additionally, autologous fat grafting was performed to enhance volume and improve symmetry on the affected side.

**Discussion:**

Poland’s syndrome encompasses a spectrum of chest wall and limb anomalies. Breast reconstruction in such patients is challenging, especially in the context of rib defects. Traditional expander or implant-based techniques are not always feasible. Our reconstructive approach preserved nipple-areola complex (NAC) sensation and glandular tissue, with emphasis on both aesthetic restoration and preservation of physiological function.

**Conclusion:**

Reconstructive planning for Poland’s syndrome should be individually based on anatomical constraints and patient expectations. In selected cases, autologous, minimally invasive techniques may yield optimal cosmetic results.

## Introduction

Poland’s syndrome, first described by Sir Alfred Poland in 1841 during the autopsy of a 27-year-old male, is a rare congenital anomaly marked by unilateral absence or hypoplasia of the pectoralis major muscle, often accompanied by defects in the ipsilateral upper limb and thoracic wall.^1^ Over time, various surgical techniques have been proposed to address the broad phenotypic spectrum of this syndrome, which ranges from mild hypoplasia to complete aplasia of ribs and muscles. The prevailing hypothesis attributes the underlying cause to embryologic disruption of the subclavian artery and its branches during the sixth week of gestation.^2^

For a definitive diagnosis of Poland’s syndrome, the coexistence of pectoralis major muscle deficiency with at least one additional anomaly - including rib abnormalities, breast hypoplasia/aplasia, or upper extremity malformations - is generally required.^3^ The condition is reported to be approximately three times more common in males and has an estimated incidence of 1 in 30,000 live births.^4^

In female patients, cosmetic concerns dominate the clinical picture, primarily due to underdevelopment or absence of the breast and nipple-areola complex (NAC). While reconstructive surgery in these cases is often sought for aesthetic reasons, surgical planning must also consider potential complications, and patient preferences.^5^ This report details a case of Poland’s syndrome with supernumerary and ectopic breast tissue, emphasizing minimally invasive reconstructive strategy tailored to the individual anatomy and expectations of the patient.

## Case presentation

**Fig. 1:**
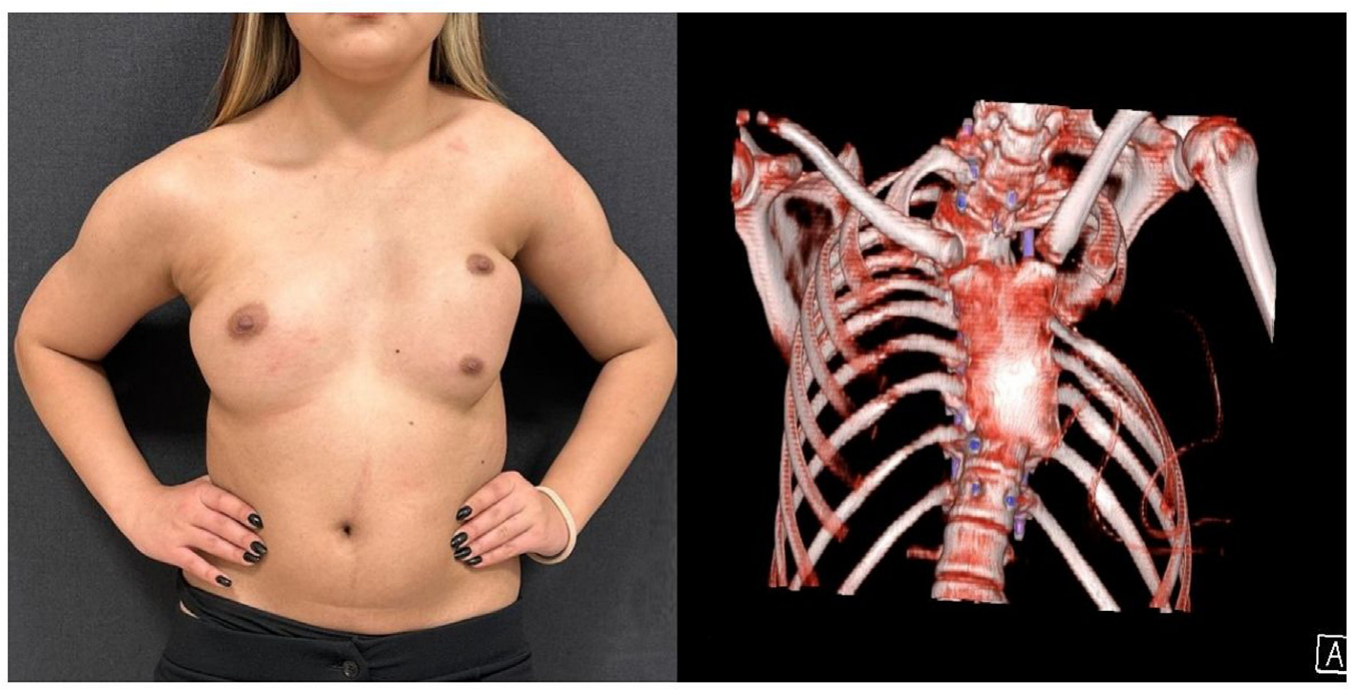
Preoperative patient picture and Preoperative 3D-CT image anterior view that shows rib agenesis.

A 20-year-old female with a known diagnosis of Poland’s syndrome presented to our outpatient clinic with concerns of left-sided breast asymmetry and supernumerary nipples. Clinical examination revealed the presence of two vertically aligned nipples on the left side and a discrete area of accessory breast tissue located approximately 6 cm lateral to the midclavicular line (Fig. 1). Notably, physical and radiographic evaluation confirmed the absence of the third through seventh ribs and their associated costal cartilages on the left hemithorax (Fig. 1). Additionally, mild shortening of the ipsilateral clavicle and humerus, hypoplasia of the chest wall and hypoplasia of pectoralis major, pectoralis minor, serratus anterior and latissimus dorsi muscles supported this diagnosis.

According to the classification by Foucras et al., the patient was categorized as having a severe phenotype. The absence of supportive rib structures and a tight skin envelope significantly limited traditional reconstructive options, such as tissue expander placement. Furthermore, although the breast tissue was malpositioned, it exhibited substantial volume, offering an opportunity for autologous reshaping.

A single-stage reconstruction was planned. Surgical design included: [1] excision of the ectopic NAC and accessory breast tissue, [2] elevation and inferior transposition of the more developed glandular component on a centrally based dermoglandular pedicle, and [3] repositioning of the NAC using a superior pedicle mastopexy technique. Preoperative markings were performed accordingly.

**Fig. 2:**
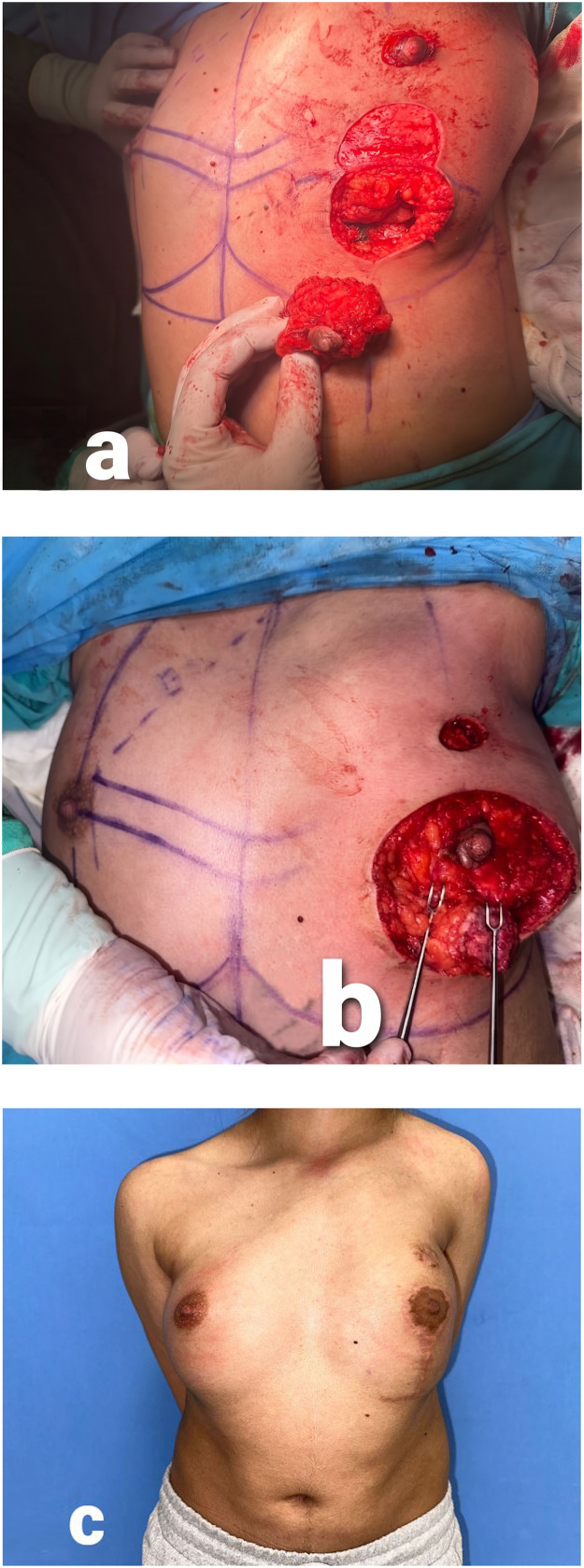
(A) Surgical planning and mastopexy incision with excision of ectopic breast tissue and nipple–areolar complex. (B) Intraoperative view showing inferomedial mobilization of the parenchymal pedicled flap. (C) Postoperative result at 6 months.

The procedure was initiated with the mastopexy incision and de-epithelialization of the mastopexy area that included the inferior ectopic NAC. The NAC and ectopic breast tissue was excised en-block from this area (Fig. 2). Through a periareolar incision, the breast parenchyma was elevated from the overlying skin envelope. The parenchyma was then mobilized off the chest wall to the level of the medial perforators, preserving a medially based pedicle to fashion a parenchymal flap. The flap was rotated clockwise and transposed inferomedially, thereby achieving the planned position of the nipple–areola complex and reconstructing the breast mound (Fig. 2). To assess perfusion intraoperatively, Indocyanine Green (ICG) angiography was performed using a SPY fluorescence imaging system, which confirmed adequate vascularization of the flap.

No intraoperative or early postoperative complications were observed. Sensory function of the NAC was assessed using the Semmes-Weinstein monofilament test preoperatively and at follow-up, demonstrating no significant change. One year after the initial surgery, the patient underwent a minor revision for additional fat grafting and scar refinement.

## Discussion

Poland’s syndrome presents a complex reconstructive challenge due to its variable clinical manifestations, including chest wall deformities, breast and NAC hypoplasia or aplasia, and underlying musculoskeletal anomalies.^3^ In severe phenotypes, particularly with absent costal cartilages, conventional options such as tissue expanders or implants are limited due to the lack of structural support and risk of complications including implant migration, capsular contracture, or impaired pulmonary function.

In our case, the absence of ribs in the breast footprint precluded the use of prosthetic devices, which could have applied direct pressure on the lung. Moreover, the existing glandular volume allowed for a conservative and autologous approach. Instead of introducing foreign material or resorting to more invasive flap reconstructions such as the latissimus dorsi or TRAM flaps, we utilized a centrally based parenchymal flap combined with fat grafting to achieve symmetry while preserving tissue integrity.

Breast deformities in women with Poland’s syndrome can range from mild hypoplasia to aplasia. Foucras et al. developed a classification for this syndrome which presents a wide spectrum of clinical features. This classification categorizes patients into mild, moderate, and severe types according to the level of breast asymmetry, the relationship of the nipple-areola complex with the inframammarian fold, and the level of hypoplasia of the pectoralis major muscle (Table 1).^4^

**Table 1:** Expanded classification of Poland’s Syndrome as defined by Foucras et al.

Type	Breast	Nipple-Areola	Pectoralis Major	Thoracic Deformity
**Mild**	Hypoplasia, asymmetry	Small, elevated	Hypoplasia	No Deformity
**Moderate**	Significant hypoplasia, aplasia, asymmetry	Hypoplastic or absent	No sternocostal head	Moderate
**Severe**	Total aplasia	Hypoplastic or absent	Total absence	Severe

The preservation of nipple sensation and glandular tissue was prioritized given the patient’s young age and nulliparous status. Sensory testing confirmed maintained function of the NAC postoperatively. Additionally, the use of ICG angiography allowed for intraoperative assessment of perfusion, enhancing surgical safety and optimizing flap viability.

While there is no established increased risk of breast cancer in patients with Poland’s syndrome, some literature suggests a possible association, warranting continued surveillance. Our reconstructive approach preserved nipple-areola complex (NAC) sensation and glandular tissue, with emphasis on both aesthetic restoration and preservation of physiological function.

Our approach emphasized individualized planning, minimal invasiveness, resulting in a successful aesthetic and reconstructive outcome tailored to the patient’s anatomical and psychosocial needs.

## Conclusion

This case highlights the feasibility of performing breast reconstruction in severe Poland’s syndrome without the use of implants or extensive flap surgery. By utilizing native glandular tissue and fat grafting, we were able to achieve symmetry and preserve nipple sensation and breastfeeding potential. Individualized, anatomy-driven planning is essential for optimizing outcomes in such complex congenital cases. Minimally invasive and autologous methods should be considered whenever viable, especially in young patients with significant reconstructive challenges.

## Funding

None.

## Ethical approval

The study includes ethical approval. But there is no reference number of the Judgement. A copy of the consent will be sent.

## Declaration of competing interest

None declared.
